# Accidental traffickers: qualitative findings on labour recruitment in Ethiopia

**DOI:** 10.1186/s12992-023-01005-9

**Published:** 2023-12-14

**Authors:** Joanna Busza, Zewdneh Shewamene, Cathy Zimmerman, Annabel Erulkar, Eyasu Hailu, Lemi Negeri, Elizabeth Anderson, Yuki Lo

**Affiliations:** 1 Department of Public Health, Environment & Society, Faculty of Public Health and Policy, London School of Hygiene and Tropical Medicine, 15-17 Tavistock Place, London, WC1H 9SH UK; 2 Department of Global Health and Development, Faculty of Public Health and Policy, London School of Hygiene and Tropical Medicine, 15-17 Tavistock Place, London, WC1H 9SH UK; 3 Population Council, Heritage Plaza, Bole Medhaneialem Road, Addis Ababa, 18609 Ethiopia; 4 The Freedom Fund, Lighterman House, 30 Wharfdale Road, London, N1 9RY UK

**Keywords:** Irregular migration, Trafficking, Ethiopia, Labour recruitment, Domestic workers, Middle east, Qualitative research

## Abstract

**Background:**

The growth of labour migration and associated risks of human trafficking and exploitation remain significant global human rights and health challenges. There is increasing policy interest in addressing structural determinants of adverse migration outcomes such as migrants’ use of informal employment recruiters. In Ethiopia, “safe migration” policies have introduced regulations for registered private employment agencies and penalties for anyone else placing migrants into work overseas. Yet migrants continue to use informal facilitators who are often demonised as traffickers without evidence of their motivations, experiences or perceptions. We conducted qualitative interviews with 28 informal facilitators as part of a study into how recruitment practices shape risks for female migrants seeking domestic work in the Middle East and Gulf States. We present the realities of irregular recruitment on the ground, and how these practices are affected by policies that dichotomise recruiters into legal/safe and illegal/unsafe categories.

**Results:**

We identified four main themes. First, arranging migration from rural areas differs from in the capital, Addis Ababa, where laws and regulations originate. Outside Addis Ababa, registration was difficult for facilitators to arrange, with little incentive to do so due to its lack of importance to prospective migrants. Second, the ability to circumvent legal requirements was considered an advantage of informal facilitators because it reduced costs and expedited migrants’ departure. Third, facilitators did not work alone but operated in long “chains” of diverse actors. This meant migrants’ safety was not determined by any given individual, but spread across numerous people involved in sending a migrant abroad, some of whom might be registered and others not. And finally, facilitators did not believe they could realistically safeguard migrants once they were outside of Ethiopia and working under different laws and employers.

**Conclusions:**

Findings from this study add to a growing body of work demonstrating the diversity of people involved in the migration process, and consequent oversimplification of popular policy solutions. A more effective approach might be to constructively engage informal facilitators and identify ways they could assist with referring migrant workers to registered agencies and safe employment, rather than criminalising their participation.

## Background

The growth of labour migration as a global phenomenon continues to attract attention and concern, particularly people’s risk of coercion and exploitation. The International Labour Organization (ILO) reported 49.6 million victims of modern slavery for 2021, comprising forced labour (27.6 million) and forced marriage (22 million). In addition to being a gross violation of human rights, human trafficking is a well-recognised determinant of poor physical, sexual and mental health outcomes [1–8]. Among an estimated 169 million migrant labourers globally [9], the number who find themselves in “dirty, dangerous and demeaning work” [10] remains unknowable, although its negative implications for health are clear [11, 12]. Taking a public health approach to the harm caused by human trafficking and labour exploitation suggests the need to intervene “upstream” to address their structural determinants [13, 14]. Yet despite decades (even centuries [15]) of efforts to curb trafficking, there have been few insights into effectiveness of most approaches [16–18] and, conversely, there has been evidence of damage caused by heavy-handed anti-trafficking measures, often motivated by political and social concerns related to migration and/or sex work, more broadly [19–23]. Contentious debates about terminology used around human trafficking and forced labour have influenced policy approaches to labour migration, often in an effort to catalyse action through emotive language. For example, there has been a substantial shift, particularly in high-income countries, to the use of “modern slavery” to refer to exploitative labour, but which also overlaps with the means of procuring exploited workers [24]. This terminology has been criticised by scholars who contend that the use of broad language leads to conflation between forced labour, human trafficking, labour exploitation and sex trafficking and over-simplifies the nuances within human rights frameworks [25].

A policy trend gaining increasing traction is to focus on the practices of recruiters and brokers in source countries, with the goal of reducing exploitation by regulating those who assist migrants to seek work abroad [26, 27]. Legislative frameworks for “safe recruitment” create licensing or registration mechanisms, one consequence of which has been the dichotomisation of “legal” and “illegal” recruiters. The former group is assumed to be legitimate and “safe” and the latter group to be traffickers who put potential migrants at risk. However, in practice, this has proven to be a simplistic characterisation that may not reflect how prospective migrants view the facilitators on whom they rely for recruitment, nor the actual practices of recruiters [28]. While analysis of data from victims of trafficking has indeed identified high rates of use of “third-party employment recruiters”, comparable data on irregular migrants who have not experienced trafficking are generally not available, making it difficult to attribute exploitation or trafficking to specific types of facilitators [29].

There has been substantial criticism of this use of binary language and undue emphasis on the recruitment process. For example, Pocock et al. (2021) describe how migrants’ own family and community members become “accidental traffickers” if policies define all migration facilitators operating outside the law has human traffickers, even where they are unaware of the illegality of their role or practices [30]. United National Palermo Protocol defined human trafficking as “the recruitment, transportation, transfer, harbouring or receipt of persons, by means of the threat or use of force or other forms of coercion, of abduction, of fraud, of deception, of the abuse of power or of a position of vulnerability or of the giving or receiving of payments or benefits to achieve the consent of a person having control over another person, *for the purpose of* exploitation” [31] (emphasis added). Many anti-trafficking strategies appear to assume exploitation is the motivation for all informal facilitators [32, 33].

In this paper, we examine the realities of “labour migration recruitment” on the ground, and how these practices are affected by policies that divide recruiters into two mutually exclusive categories of legal/safe and illegal/unsafe. We use the example of Ethiopia, which has experienced several policy shifts related to regulation of overseas employment agencies and informal facilitators in recent years, and has adopted a “safe recruitment” approach. In particular, we consider the perspectives and experiences of people who might fall into the category of “accidental traffickers” because they are involved in arranging overseas migration outside of the legal framework, who we refer to throughout this paper as *informal facilitators* (as compared to officially registered recruiters). Voices of informal facilitators are rarely included in research around labour migration, particularly where their actions may be deemed criminal under initiatives to prevent labour exploitation or human trafficking.

### Ethiopian context

Hundreds of thousands of Ethiopians are estimated to reside in the countries of the Middle East and Gulf States, many in precarious situations due to irregular forms of migration out of Ethiopia, undocumented or illegal status in the destination countries, and poor legal protections or access to redress in cases of exploitation and abuse [34]. Ethiopia is a major “source” country for female migrants entering the domestic labour market in the Middle East (primarily Saudi Arabia, Kuwait, Bahrain, United Arab Emirates and Lebanon) [35, 36]. Studies conducted among returnee Ethiopian domestic workers have documented extensive negative mental and physical health outcomes resulting from mistreatment, neglect, and occupational harms [35, 37–40].

In response to growing media coverage of abuse of Ethiopian migrants, the Ethiopian government imposed a ban on all labour out-migration between 2013 and 2016, although many Ethiopians continued to depart illegally [41]. The ban was revoked in favour of national “safer migration” policies, including the Overseas Employment Proclamation No. 923/2016 and bilateral labour agreements signed with destination governments including Saudi Arabia, United Arab Emirates (UAE), Jordan, Kuwait and Qatar, with negotiations opened for Lebanon and Bahrain. The Proclamation includes strict licensing requirements for private employment agencies (PEA), criminalisation of non-registered recruiters, and establishment of requirements for migrants such as minimum age and pre-departure training to procure a Certificate of Competence (CoC). To operate legally, PEA must register with the Ministry of Labour and Social Affairs (MoLSA), which requires operating capital of 1 million Ethiopia birr (USD $45,800 in 2016; USD $18,100 in 2023) with an additional deposit into a workers’ protection account set at USD $100,000 (approximately 5.5 million Ethiopian Birr in 2023). PEA are responsible for ensuring compliance with pre-departure eligibility requirements for migrants and labour standards in overseas placements, which can be made only in countries with bilateral agreements with Ethiopia [42]. In 2020 the revised Prevention and Suppression of Trafficking in Persons and Smuggling of Persons Proclamation (No.1178/2020) established a task force with presence in all regions and districts (*woredas*) of the country that is responsible for identifying and prosecuting traffickers, as well as assisting victims of trafficking. In addition to targeted police action, community members can report suspected traffickers to the local task force. Awareness-raising campaigns warning Ethiopian communities of risks associated with illegal labour migration (i.e., other than through registered PEAs) have been widely implemented. The Proclamation specifies 7 to 15 years of imprisonment and a fine of 20,000 to 100,000 Ethiopian birr ($360 to $1800 in 2023) for labour trafficking and adult sex trafficking, increasing to 10 to 20 years’ imprisonment and a fine of 30,000 to 100,000 Ethiopian birr ($540-$1800 in 2023) for trafficking committed by organizations licensed to conduct domestic or foreign employment [43].

As a result of these and other measures, in 2023 Ethiopia was “upgraded” to Tier 2 in the US Trafficking in Persons annual report [44], and specifically praised for legal prosecution of traffickers. The report reads “The government increased its use of the 2020 anti-trafficking proclamation and reported sentencing data… which reflected adequate penalties for convicted traffickers involving significant prison terms” [45]. A specified weakness of Ethiopia’s measures, however, also related to criminalisation of informal facilitators, as the TIP report goes on to state that “Despite reports of fraudulent labour recruiters regularly recruiting and exploiting Ethiopians seeking employment abroad, the government did not report efforts to hold fraudulent labour recruiters criminally responsible” [45].

We have previously documented reasons for migrants’ continued use of informal facilitators and “illegal” routes out of the country despite exposure to information campaigns and changes in policy [46]. Migrants’ motivations for relying on informal facilitators (vs. formal agencies) included the relative ease and speed of migrating through informal channels, a pervasive belief that registered agents do not reduce risks of migration (determined by conditions in the destination country), and difficulty differentiating between licensed and unlicensed agents. Related to the latter, we and others documented ways that some registered recruiters operate “under the law,” for example by placing women into employment without eligibility checks or requiring pre-departure training, or by charging placement fees [35, 46]. Thus a dichotomised view that formal, registered recruiters were “safe” while informal, unregistered facilitators were “unsafe” did not accurately reflect the experiences and perceptions of prospective migrants, returnees, or their families and other community members in Ethiopia – even while official recruiters continued to be actively promoted by Ethiopian authorities and international bodies [34].

To complement these previous findings, we explored the views of informal facilitators themselves, whose experiences and insights are often absent from the literature, and yet bear the brunt of policies that categorise them as “traffickers” – often leading to criminalisation and prosecution. We use empirical data from Ethiopia to highlight the disconnect between policy aims and interrelationships between prospective migrants, informal facilitators, and registered recruiters within a complex and diffuse web that make up the “necessary infrastructure for labour migration” [32]. Our aim is not to criticise policy efforts to monitor and regulate migration recruiters, but rather to broaden the “safe migration” approach and offer insights into how it might take better account of the diverse ways labour migration recruitment occurs.

## Methods

The data presented in this paper are drawn from a larger study conducted in Ethiopia between 2018 and 2020. The Meneshachin (“our departure”) study was conducted in partnership between the London School of Hygiene and Tropical Medicine, the Population Council, and The Freedom Fund with funding from the U.S. Department of State. This qualitative research sought to inform future responsible recruitment initiatives by generating knowledge about the realities of the current policy environment. As part of the study, we examined the roles of informal migration facilitators engaged in recruiting women and girls for domestic labour migration along the Ethiopia. This work focused on the Middle East corridor and the way in which facilitators and prospective migrants and their family members perceived the relative benefits and risks of choosing irregular means to seek employment abroad. For this paper, we draw on data collected from informal facilitators.

### Study sites

We conducted fieldwork in two well-known source locations from which individuals migrate to the Middle East and Gulf states: Hadiya Zone (Southern Nations, Nationalities and Peoples Region) and Bahir Dar town and its surroundings in Amhara Region. Both locations are major transport hubs (and therefore likely locations for facilitators). Bahir Dar was also selected due to its relative stability at the time of data collection, despite previously having been affected by the conflict between Ethiopian Federal forces and the breakaway Tigray region.

Before fieldwork started, a mapping exercise was conducted in both sites to identify “feeder towns” from which women travel to arrange onward migration. Lists of local stakeholders and key informants were compiled, and informal conversations held to triangulate information about the origin locations of female migrants. In Hadiya, it was easier to identify two small towns considered to be migration “hotspots”, namely Soro and Anilemo, which are known to have high numbers of women seeking domestic work in the Middle East corridor. Anilemo is primarily Muslim and located around 50 km from Hossana (capital of Hadiya Zone) on a main road. Soro is closer to Hadiya (40 km) but without good road access. Most residents of Soro are Christians. In Bahir Dar (Capital of Amhara region), it was not possible to select “feeder towns” because according to key informants and local stakeholders demand for migration was similarly high across the different *woredas* (districts). Four different urban sites (sub-cities) were thus selected for fieldwork, located in different directions from central Bahir Dar.

### Study participants and data collection

Informal facilitators in both sites were recruited through the use of locally-based Population Council research staff members’ community networks, followed by “snowball sampling” (word-of-mouth contacts from each identified facilitator). Recruiting unlicensed facilitators proved very challenging since they fear possible repercussions if identified. Overall, we experienced a 50% refusal rate (28 refusals out of 56 requests for interview), and participant recruitment proved easier in Hadiya (26 agreed out of 45) than Bahir Dar, where only two of eleven identified informal facilitators agreed to be interviewed.

Well-trained qualitative researchers collected data at both sites. Interviews were conducted in local languages (Amharic and Hadiyissa). Interviews lasted approximately 60–120 min and most were conducted face-to-face at locations convenient to the respondent (e.g., in their homes, shops, café or offices). Some facilitators preferred anonymous interview locations where they felt they were “hidden” from the public and legal authorities (e.g., villages further out of the town, rooftop terraces of hotels). The study used semi-structured qualitative interview topic guides that asked facilitators to describe how they are in contact with potential migrants, what support they provide, relationships with other recruiters, and their capacity to protect migrants both during departure and once in destination countries. Ten facilitators refused to have their interviews digitally audio-recorded and only notes were taken. All recorded interviews and notes were transcribed, translated into English, and entered into NVIVO software for analysis.

### Analysis

The first two authors led thematic analysis. Analysis started with data familiarisation, where all the transcripts were read at least twice, followed by coding using NVIVO. A coding framework developed earlier in the study was applied to the data, with newly emerging themes specific to the experience of informal facilitators added through team discussion. The lead Ethiopian researcher conducted frequent data checking by returning to the recorded audio interviews to listen to the original Amharic to optimise accuracy of translation and provide quality control for transcriptions. A research consultant employed by the Population Council conducted the same data checking process for audio-recordings in the Hadiyissa language. All researchers held discussion meetings during the analysis process to reflect on their initial thoughts on the data set and agree on the emerging themes to help the accurate interpretation of participants’ responses.

### Ethical considerations

All respondents provided either written and/or verbal informed consent, consistent with the study protocol. When respondents did not want to sign a consent form, they were asked to provide verbal consent on record, using digital audio-recording (which was then turned off for respondents refusing recording of the full interview). All respondents received a comprehensive study information sheet to emphasise that any information they provided would remain anonymous, that they could terminate the interview and withdraw their consent at any time, and that any data shared in reports or other research outputs would be anonymised to prevent identification of respondents or individuals they may have named. We did not ask respondents directly whether they were involved in illegal activity (beyond facilitating migration without being registered), but phrased questions to reflect practices in which “people like you” might engage. Ethical approval was obtained from LSHTM (reference number 19127) and the Ethiopian Society of Sociologists, Social Workers and Anthropologists (ESSSWA) in Ethiopia (reference number ESSSWA/L/AA/0449/20).

## Results

Table 1 provides working definitions for types of migration intermediaries in Ethiopia. These categories were identified during analysis to address the challenges of working with raw data collected in 2 Ethiopian languages. It was not possible to accurately capture in English all the nuanced differences in terms applied to the many types of people who assist migrants; furthermore, even in the same language, terms were not always used consistently. As a result, we have made the decision to use the following definitions systematically throughout the text, including in translation of direct excerpts from interview transcripts.

**Table 1:** Working definitions of intermediaries of Ethiopian women’s migration into domestic work abroad

Term	Definition
Facilitator	General term for any person engaged in assisting women migrating from Ethiopia to countries of the Middle East for domestic labour. Sometimes specified as either a formal facilitator or informal facilitator, which are terms corresponding to recruiter and broker, respectively. Facilitators usually have other forms of employment and do not rely on migration as their main source of income.
Recruiter	A person for whom arranging migration is the main form of employment, and who works alone or as part of an agency, and is responsible for the final stage of migration, i.e. departure from Ethiopia and placement with an employer abroad. Community members often use this term to suggest they believe the recruiter has a formal or legal role (i.e. is registered), although they usually have no way to verify this.
Broker	Someone who is considered an intermediary between potential migrants and other facilitators or recruiters. The term is most frequently used to refer to individuals operating at the local level who do not have contacts abroad or any form of registration. Their role is to find and refer women to others who take responsibility for migration logistics, although the broker may assist with some early preparations.

Twenty-eight informal facilitators were interviewed, 26 of whom were recruited from Hadiya. Four facilitators were female, all in Hadiya. While facilitators reported knowing that registration was a legal requirement for placing domestic workers into work opportunities abroad, they believed this prerequisite was untenable and unpopular with migrants, their families, and facilitators alike as well as an insufficient means to protect migrants. They also displayed confusion around exact stipulations of the law regarding whether an informal *association* with registered PEA meant they were operating legally (e.g. if they transferred local migrants to a registered PEA in Addis Ababa).

We identified four main themes demonstrating facilitators’ engagement with or avoidance of Ethiopia’s legal framework for migration. First, rural areas and regional cities were considered to be different from the capital, Addis Ababa, where laws are made and regulations are managed. Outside Addis Ababa, facilitators felt registration to be both difficult to arrange and not prioritised by prospective migrants, disincentivising their compliance. Second, facilitators’ ability to circumvent legal requirements was seen to be an important advantage they offered prospective migrants, who wanted to expedite their departure and reduce costs and bureaucracy. Third, accountability for migrants’ safety was difficult to situate with any given individual when numerous and diverse types of people were sequentially involved in sending a migrant abroad. And finally, facilitators did not believe they could realistically safeguard migrants once they were outside of Ethiopia and assumed that the responsibility for migrants’ safety lay with licensed agencies to which they claimed to link migrants.

### Rural needs versus urban policy

At the time of data collection, our study found there were no licensed PEA located at the regional level or zones (one administrative step down from regional government) in either field site. Respondents reported that all registered agencies were in Addis Ababa, despite the fact that both Hadiya and Bahir Dar are known to be major migration “hot spots” in the country. One barrier to obtaining a PEA licence related to administrative difficulties, as described by a female facilitator who had considered the option:

From highest to lowest levels of government there are challenges to get a license to engage in this facilitation process. …They [government] makes the process to get a license complicated and difficult to complete. The bureaucracy is very tedious … and they are not happy to give the license for facilitators in a short period of time [quickly] even though we fulfill the requirements. (Facilitator 2, Hadiya, Female).

Most facilitators described referring rural migrants on to registered recruitment agencies in Addis Ababa, doing this informally because there is no way for them to obtain legal recognition for an intermediary role. These facilitators explained that community members therefore think they are part of the regulated system.

I am in informal facilitator, but people in this community consider me as a formal facilitator (Facilitator 22, Hadiya, male).

My role is supplying women to a certified person in Addis Ababa. … I call a legal person who works in Addis Ababa, he arranges everything including food and a place to stay [prior to flight]. … There should be agencies locally. For example, we don’t have legal agencies in Hosanna. … I can do nothing different. It [operating informally] is a risk [that I take] (Facilitator 3, Hadiya, male).

One facilitator interviewed in Hadiya claimed to be registered, although he did not provide any documentation to this effect, and other facilitators suggested this was unlikely. Later in the interview, he described how he processes migration as far as Addis Ababa, after which a separate agency takes responsibility for sending women abroad and placing them into domestic work; it is possible (but impossible to verify) that this second agency has a license, and thus the local facilitator believes that he is also registered. In reality, it is probably very difficult for either prospective migrants or facilitators to be sure of the legal status of any agency located in Addis Ababa, and they are likely to trust what they are told by the agency staff.

It would be difficult for prospective migrants to seek registered recruiters in Addis Ababa, not only due to the distance and cost of travel, but because they prefer to rely on personal networks and existing relationships of trust within their rural communities. For example, three of the four female informal facilitators who participated in this study reported being returnees themselves. Some remained in contact with former employers abroad, using these families to identify others who required Ethiopian domestic workers. They also relied on word-of-mouth recommendations within their own communities to fill positions with prospective migrants from their own community networks.

Whenever my former employers … asked me to bring domestic workers for them, I tell them that I have got relatives and neighbours that I know and who are in need. They tell me to bring them a housemaid and I will ask them to cover their visa, ticket, and transportation expenses as well as their clothing expense, and they will take them. (Facilitator 4, Hadiya, Female).

One woman referred to an “office” in the (unspecified) destination country with which she is affiliated, and that “friends” there are responsible for picking up newly arrived Ethiopian migrants and taking them to their employers. These staff members also handled any requests for a change in contract/post from either the domestic worker or employer, which is contractually permitted within 3 months before finalising employment. These colleagues are later described to be Arabic-speaking Ethiopian nationals.

My friends are in the office, so when the women arrive, they will pick them up, hire them, change their house if they are not comfortable, and arrange some money [salary payment] with the Arabs and send us some money. (Facilitator 21, Hadiya, female).

In situations in which the facilitator has direct contacts in the destination country, rural migrants can avoid liaising with PEA in Addis Ababa altogether, and prioritise the facilitator’s personal connection over assurances offered by agents whom they do not know or necessarily trust. Furthermore, before a migrant travels to Addis Ababa airport, the main tasks need to be completed at the local level by informal facilitators, including obtaining ID from the local kebele office (village civil authority), applying for a passport, and arranging transportation and accommodation in district towns or regional capitals, as noted by one facilitator:

The most significant assistance I provide to migrants is for obtaining a kebele identification card and passport (Facilitator 1, Hadiya, male).

### Circumventing regulations as unique selling point

In addition to offering personal contacts and familiarity with local systems, informal facilitators provide services *outside* the parameters of the Overseas Employment Proclamation. Respondents explained how migrants wanted to leave Ethiopia as soon as possible once they decide to seek employment abroad, often without even knowing to which country they would go. Many seek to keep their costs as low as possible.

Some migrants travel overland from Ethiopia into Sudan or Djibouti and continue by boat, via Yemen, a route recognised to be especially dangerous [47]. From Yemen, migrants continue on foot or by vehicle. The overland route is less common for female migrants seeking domestic work, but may have increased due to COVID-19 travel restrictions that limited international air travel options. The illegality of overland labour migration has been well-publicised throughout Ethiopia, and thus anyone seeking this option must rely on informal facilitators with good connections.

I have colleagues [government officials] who work with me by getting involved in different activities. For example, I deal with police officials who assisted during border crossing to easily pass the migrants (Facilitator 23, Hadiya, male).

Collaboration with authorities is particularly important for overland travel, as borders remain heavily monitored.

We are working very closely with the police force and local administrators. You know, if you don’t have a very close person in the local and lower administrative hierarchy you cannot travel an inch with 20 migrants in your hands, aiming to cross borders. Likewise, friends in Sudan have their own close contacts to make travel fast and support migrants to reach their destination countries (Facilitator 27, Bahir Dar, male).

However, most women depart from Addis Ababa airport, where paperwork is more likely to be checked. Facilitators explained that prospective migrants view meeting eligibility criteria, completing pre-departure medical checks, undergoing pre-departure vocational training, and obtaining the Certificate of Competence (CoC) as barriers to overcome rather than important safeguards. As a result, informal facilitators’ unregulated status was perceived as an advantage over licensed PEA, something facilitators could capitalise on by advertising their ability to eschew regulations.

I know formally registered agencies need several documents such as CoC, certified educational documents and training documents. When travelling through informal means there is no need for such kind of certificates. They [migrants] need only money. Therefore, using informal facilitators is faster than the formal facilitators (Facilitator 28, Bahir Dar, male).

In fact, some requirements had changed between 2016 and 2020, including proof of having completed 8th grade. Awareness of efforts to streamline the migration process had largely not filtered down to community level at the time of this study, making the use of informal facilitators appear very advantageous.

Another way that facilitators can hasten the migration process is to forge required documents. Respondents described how training certificates, proof of educational attainment, and medical checks could be provided by facilitators. In addition to meeting migrants’ desire for speed, forging documents provided facilitators with an additional source of income.

If she wants to train and earn the CoC she pays 2500 birr, but if she doesn’t want to do so, we will prepare a CoC and she pays 3500 birr (Facilitator 20, Hadiya, male).

Although obtaining false documents is clearly a breach of the law, it makes it possible for the migrant to then be passed on to a registered PEA and migrate via the regular systems. Without any documents, migrants need to rely on a local facilitator to place them into employment abroad, as described by female facilitators from Hadiya who used their personal connections from their previous domestic work abroad. Once an informal facilitator has helped a prospective migrant procure her ID, passport, CoC and health certificate – whether legitimately or illicitly – she can enter the migration pathway set forth in the Proclamation if she then migrates using a PEA that does not scrutinise the authenticity of her documents or how she got them.

### Recruitment chains are long and tangled

We identified a complex web of social and professional networks engaged in moving women from their local communities to their eventual destination. The informal facilitators are, as already described, often the first point of contact. They may be sought by a prospective migrant or members of her family, although some facilitators proactively sought local women who might be interested in enlisting their services.

We directly approach them [potential migrants] based on information from different sources. We get information from her friends, families or others, for example that a particular woman has a passport and is searching for a facilitator to help her (Facilitator 8, Hadiya, male).

Once a woman has found an “entry point” into the migration process, she will then be passed on to other facilitators who take on different roles as she progresses. While local facilitators may help her obtain the kebele ID and direct her to a passport office, they may then hand her over to someone who works more regionally and organises transport to Addis Ababa, or in the case of Bahir Dar, over the land border to travel via Sudan.

They come to Hossana [from rural villages] and I will send them to Addis in a public bus. I will send the car’s plate number and their name list via the [social media platform] telegram to the facilitator in Addis Ababa (Facilitator 5, Hadiya, male).

We send migrants mainly through Metema [to Sudan] and Afar [to Djibouti] then to Yemen and then to other Arab countries. First, we collect migrants from different [rural] areas through networking with other facilitators. After collecting migrants from these agents, we directly send them to another agent in Metema, then another agent from Sudan or Djibouti receives them and sends through water transportation to another Arab country (Facilitator 27, Bahir Dar, male).

As suggested above, migration facilitation, especially overland travel, can involve a very loosely connected network of numerous actors, most of whom are not official. The local informal facilitators are often the only individuals with whom migrants have direct contact. Their subsequent links are to facilitators with whom they communicate by phone or online through platforms such as Facebook. Facilitators taking an intermediary role higher up the “chain” reported wanting to avoid detection and thus operated “behind the scenes,” relying on local brokers to make the initial introduction.

Most of the time migrant women can’t find me face to face. . most of the time our contact is by phone. . because police officials may follow us to arrest or jail us (Facilitator 24, Hadiya, male).

Both community members and facilitators themselves are unaware of how many people are in the “chain” that eventually gets migrants abroad. As a result, women value their first point of contact most, and put their trust in them, without much thought about who will take ultimate responsibility for their travel and placement.

They put their trust in the beginning with … a relative or a family or a friend, that’s how trust is built, with a person that connects migrants to a broker or agency. (Facilitator 11, Hadiya, male).

The facilitators interviewed for this study represented different links in these long facilitation chains. They described networks of facilitators that included cooperation with local authorities who support their practice and share financial benefits. For example, facilitators cooperated with religious leaders, health extension workers, all the way up the system to employees of government and business offices.

There is a strong chain of migration: officers in government offices like the immigration office, embassies, airlines, police, foreign minister and other offices violate rules and work with informal facilitators. (Facilitator 5, Hadiya, male).

For those migrants who do not go overland, eventually the prospective migrant reaches an agent in Addis Ababa, through at least two and often many more steps, many of which brought them in contact with a diverse mix of regular and irregular actors (or, indeed, regular actors operating outside the legal framework). The length and density of migration “chains” mean that migrants and their families are entirely unaware of how migration is being arranged, thus making it impossible to hold any particular individual accountable for subsequent difficulties. Facilitators themselves did not always appear to know their exact position in a “chain” nor how many links there might be.

### Duty of care not realistic

According to informal facilitators based in Hadiya Zone and Bahir Dar, migrants’ safety was ensured by using legally registered recruitment agencies for the *final* step of the journey, i.e., sending women out of the country. Overall, community-based facilitators who referred prospective migrants to someone in Addis Ababa said that these facilitators would protect migrants at destination because they believed the agencies operated according to legal requirements.

I hand them to the licensed person. . I do believe they will [protect migrants] because I work with a licensed facilitator (Facilitator 18, Hadiya, male).

Agencies that are registered are better for everything. They take responsibility to keep the migrants safe and to return them in case of accident or danger (Facilitator 3, Hadiya, male).

Whether facilitators genuinely believe (or know) that they are referring migrants to licensed agencies is impossible to verify. Only one facilitator described going through a process of verification about the status of the recruitment agency to which he sends local migrants:

My role is to go and check that agency, I see in which office it is based, if it’s real, I check if it is legal … if it is licensed by the government and if it has no problems, otherwise I wouldn’t recommend our sisters to a random agent, just for the sake of money. (Facilitator 11, Hadiya, male).

Beyond assuming that sending agents will protect migrants, the facilitators interviewed in this study did not consider themselves to be responsible for securing the safety of migrants once they had been passed further up the “chain” towards departure.

My responsibility will end after migrants reach another facilitator. ... Frankly speaking almost all informal agents do not think about responsible recruitment. Rather they are focusing on making money (Facilitator 28, Bahir Dar, male).

Furthermore, community-based facilitators did not believe they *could* assist migrants abroad. This was primarily for practical reasons, as most of the interviewed facilitators worked at local level, and either had no links in destination countries or were not able to exert any influence over them. Facilitators were unable to identify ways that they would be able to access a migrant who experienced difficulties in another country, as several explained:

It depends on her luck, and if we meet a good person, we will help, but we don’t have the confidence and the ability to take her out of the situation (Facilitator 16, Hadiya, male).

Yes, [I can protect them] until they reach Sudan, I can deliver any protection but sometimes there are uncertain situations especially from security personnel. But to speak honestly, I am afraid I cannot deliver any protection once they are out of Ethiopia. You know things are out of our control and it depends upon the strength of our colleagues working in Sudan and Saudi Arabia [Facilitator 27, Bahir Dar, male).

Moreover, facilitators know that they are operating illegally, and fear being arrested, so are therefore unwilling to draw attention to themselves by trying to intervene or alert the authorities.

Leave alone protecting the migrants, I am not able to protect myself as I am in an illegal line of business. Only the migrants themselves are responsible for protecting themselves. (Facilitator 5, Hadiya, male).

As noted above, facilitators are unlikely to assist migrants because of their limited reach beyond Ethiopia’s borders and because of the risk of making themselves known to others involved in an irregular migration process.

## Discussion

In Ethiopia, “safe migration” policies increasingly focus on the role of recruiters in putting migrants at risk of human trafficking, labour exploitation and its associated harms. Legal proclamations include both strict regulations for registered private employment agencies and penalties for anyone else who places migrants in jobs abroad. Nonetheless, migrants continue to use irregular forms of migration and rely on informal facilitators to navigate their journeys. To date, studies have rarely examined the perspectives of informal facilitators about their role in the migration process. As part of a larger study into how recruitment practices shape determinants of risks for female migrants seeking domestic work in the Middle East and Gulf States, we interviewed 28 informal facilitators based in areas known as significant “source” locations for domestic labour migrants and situated far from the capital city, Addis Ababa.

We found facilitators to be diverse and that they take on different tasks related to assisting individuals to migrate. Some facilitators were returnee migrants themselves and had personal contacts in a destination country. Others were community members who proactively sought out prospective migrants on behalf of more established recruitment agents, often based in the capital. Informal facilitators play a valued role in the recruitment process, particularly in rural areas where access to licensed PEAs is difficult if not impossible. At the time of fieldwork, there were no registered agents available in Hadiya Zone or the Bahir Dar “catchment area”, leaving prospective migrants with no realistic choice but to work outside the regulated system, at least in the preparation stages.

Facilitators and migrant workers alike did not perceive government attempts to register recruitment agents as protective but instead onerous and time-consuming. Therefore, some facilitators linked up with agents based in Addis Ababa that they claimed were licensed PEAs. However, current law prohibits registered PEAs from hiring “outreach staff” in rural areas (the ban aims to prevent aggressive recruitment of community members). Instead, migrants and their families are expected to contact agencies directly. Making contact with distant formal agencies on their own is generally untenable as rural families are unlikely to know whom to contact, how to get in touch, or afford travel to Addis Ababa to find a registered agency, reinforcing their reliance on local informal facilitators.

While restricted access to registered recruiters is one reason why migrants opt to use informal facilitators, another is the very appeal of their ability to operate outside government restrictions. Our previous research and that of others has shown that migrants prefer to reduce the bureaucratic and time burden of migration procedures [46, 48]. When eligibility requirements or pre-departure prerequisites are poorly explained, not streamlined, and inefficient to complete, migrants turn to informal facilitators to circumvent regulations, thus expediting their travel. Some facilitators in our study were able to send migrants directly to a work placement abroad, but more commonly they described being able to support procurement of ID cards and (no longer necessary) educational requirements, sometimes through illicit means. Some offered forged Certificates of Competence to falsely prove completion of pre-departure training. A recent report highlighted that these training courses are among the least well-implemented components of Ethiopia’s anti-trafficking initiatives, with long waiting times for those that are freely available through governmental institutions, while private agencies charge fees considered prohibitive by most migrants [35]. The unlicensed, illicit and easily accessible nature of informal facilitators thus becomes an advantage for some prospective migrants, further disincentivising facilitators from going through the licensing process. Those who assist migrants to depart from Ethiopia through the overland route, by definition, operate illegally. There is little doubt that their popularity increased during times when air travel was restricted, such as during the 2013–2018 ban on labour migration and COVID-19 pandemic [48, 49].

As found in previous research in Ethiopia [49], migration is a process facilitated across whole communities, including different types of respected and trusted authorities or traditional leaders. Most facilitators do not operate independently, but link together in “recruitment chains” with locally-based facilitators responsible for arranging relevant documentation and then passing migrants to other facilitators with the capabilities and contacts required for transport out of the country. The chains can be so long that facilitators themselves do not know who is ultimately responsible for placing migrants in overseas employment, a phenomenon seen in many other studies of migrant domestic workers’ recruitment journeys [32, 33] and referred to by Schapendock (2017) as the “fuzzy web of migration facilitation” [50]. These migration networks can involve a multitude of actors, including short-range taxi drivers, longer-haul transporters, hostel owners, and their local associates [28]. While most of our respondents claimed they linked their clients to registered agents in Addis Ababa, they did not have any means to verify this nor were they necessarily certain. Furthermore, they seemed to hope optimistically that legality of the final “sending” agency might confer legality on their own practice, even as they admitted that they knew they were working outside the official system. This makes the distinction between “legal” and “illegal” migration difficult to identify in practice. This perceived ambiguity also suggests that some facilitators may not intend to operate outside the law and would have little reason to believe they could be considered ‘human traffickers’ or involved in forced labour.

Facilitators in Bahir Dar and Hadiya Zone placed responsibility for ensuring migrants’ safety on those who arrange travel to the final destination. While this could be an attempt to deflect accountability away from themselves, informal facilitators also raised the valid point that they have no control over work conditions in destination countries. Moreover, findings from our previous study indicate that migrants also believe that their facilitator has little obligation to them once they leave the country [46]. Administratively, facilitators have limited reach, because they cannot be hired by PEAs as field staff, and, like migrants themselves, they must rely on what they have been told by the agents with whom they work. At the same time, many try to reduce their contact with migrants out of fear of potential prosecution and legal penalties. As suggested by our low response rate among informal facilitators, they feel reluctant to admit to this work. This makes it difficult for migrants who confront difficulties to find them for assistance or redress.

Evidence suggests that migrants in the Middle East and Gulf States, regardless of country of origin and including both those who used regular and irregular means of migration, experience significant exploitation and abuse due to inhospitable, extremely discriminatory environments for low-wage workers, few or no support and referral resources, insufficient legal protection and little to no redress [35, 48, 51, 52]. Again, the often complex network that helps a migrant get from their home to a job seems to blur the assumptions that “legal = safe” and “illegal = unsafe”, which is the rationale driving many “safe migration” policies. These ambiguities simultaneously highlight the serious challenges that will arise when trying to implement and enforce recruiter registration regulations.

Our findings on the inherent limitations of local recruitment agency regulations indicate the importance of renewed efforts to strengthen regulation of job placement agencies *based in destination locations*, which should be accompanied by improved government responses to abuses by employers and placement agencies. Other studies have also highlighted the importance of supporting the rights of migrant workers *where they work* rather than prior to their departure. In addition to concerted state responses in destination countries, including bilateral diplomatic agreements, perpetrator prosecutions and genuine compensation schemes, initiatives to protect migrant workers might also include accessible helplines, local migrant service organisations, and soft support via networks of migrants from their home countries [17, 53]. These types of formal support services should be a central destination state investment alongside state-funded initiatives to improve ethical job placement and worker safety in their countries. There is little logical reason to believe that improvements in work conditions will be achieved simply via improving enforcement of regulated pre-departure actors in source countries.

Findings from this study also add to the growing body of work demonstrating the breadth and diversity of people involved in the migration process, and the concurrent oversimplification of potential policy solutions [54]. As attention to human trafficking increased, this criminal-justice framework has erroneously suggested that the types of labour recruitment practices observed in this study are motivated solely by the recruiters’ desire to exploit the migrants enlisting their services [30, 55]. Many studies have highlighted that family members, friends, fellow migrants, neighbours, returnees and professional “recruiters” all make up part of the global “necessary infrastructure of migration” – whether or not they operate within sanctioned regulations [32, 33, 49, 54]. While the “labour exploitation continuum” is an understood concept [56], where poor work conditions can range from the demeaning to extreme exploitation and various forms of modern slavery, there is less tolerance for a continuum of migration-related roles, spanning informal and formal actors to the criminally motivated human trafficking. A more effective approach could consider how to regulate and monitor recruitment practices while identifying how informal facilitators might be engaged as allies to assist in referring migrant workers to safe jobs rather than criminalising their participation.

Strengths of our study include our inclusion of informal facilitators operating in different migration environments in Ethiopia, a country that sends among the highest number of migrant domestic workers to the Middle East and Gulf States. We were able to reach both male and female facilitators, including those who are former migrants, those with direct contact with migrants and more remote “middlemen”, as well as those involved in the relatively rare and highly secretive “overland” route out of Ethiopia. These rarely produced findings complement evidence drawn from the perspectives of returnee migrants, many of whom are purposively selected into research for having been victims of trafficking [40, 57–60]. We also built on prior research into recruitment conducted among returnees, prospective migrants, family members or current or former migrants, and key stakeholders in the same study sites [46]. This foundation allowed us to iteratively develop our research questions and data collection tools to triangulate previous findings with the neglected voice of facilitators (referred to by Lindquist et al. as opening the “black box” of migration research [61]).

Limitations of this study include difficulties in accessing informal facilitators and a high refusal rate, which is likely to have introduced bias into our findings. It is possible that those who agreed to be interviewed were less likely to be involved in the most stigmatised and criminalised forms of migration recruitment, including coercion and trafficking. We also interviewed a much higher number of respondents from Hadiya Zone than Bahir Dar (where identifying informal recruiters proved more difficult and consent rates were lower). As with all qualitative research, findings cannot be generalised to other contexts nor considered representative of all facilitators in Ethiopia, which is an exceptionally diverse geographically, culturally and linguistically. Nonetheless, our study sheds light on an under-researched area with relevant implications to broader debates on policy measures and intervention strategies to reduce the risks of extreme exploitation and long-term harm among aspiring migrant workers.

## Conclusions

Labour migration is an age-old phenomenon and is a persistent feature of our globalised world. But, as calls for action against human trafficking have risen, economic migration has become entangled with international outrage about modern slavery. Avoiding this conflation between perpetrators of human trafficking and labour intermediaries in policy-making will be increasingly important as climate change leads to greater population mobility due to diminishing viability of traditional livelihoods [62]. Estimates from 2022 suggest that approximately 32.6 million people were displaced due to the effects of climate and environmental events [63] and a recent report highlighting Ethiopia’s vulnerability to loss of land productivity specifically predicted a consequent rise in population-level intent to out-migrate [64]. Labour intermediaries will remain an integral part of the migration process. Existing anti-trafficking debates should avoid binary and emotive language, but rather embrace the nuances of international labour migration and seek to find context-specific ways to address the local migration landscape. Using blunt policy instruments that demonise informal facilitators risks pushing them to operate further under the radar. Informal facilitators provide a service seen as essential to many communities, particularly in rural areas, and are typically trusted by them, including women who are seeking work opportunities abroad. Government and civil society organisations could engage more deliberately with facilitators as key messengers in order to provide prospective migrants with more accurate advice and reduce the risk of deception and labour abuse.

## Data Availability

The datasets generated and/or analysed during the current study are not publicly available due to the sensitive nature of the topic and disclosure of illegal practices. Anyone who would like to access the raw data should contact the first author directly to discuss potential use.

## References

[1] Zimmerman C, Kiss L (2017). Human trafficking and exploitation: a global health concern. PLoS Med.

[2] Decker MR, McCauley HL, Phuengsamran D, Janyam S, Silverman JG (2011). Sex trafficking, sexual risk, sexually transmitted Infection and reproductive health among female sex workers in Thailand. J Epidemiol Commun Health.

[3] Oram S, Stöckl H, Busza J, Howard LM, Zimmerman C (2012). Prevalence and risk of Violence and the Physical, Mental, and sexual health problems Associated with Human trafficking: systematic review. PLoS Med.

[4] Richards TA (2014). Health implications of human trafficking. Nurs Women’s Health.

[5] Turner-Moss E, Zimmerman C, Howard LM, Oram S (2014). Labour Exploitation and Health: a Case Series of men and women seeking Post-trafficking services. J Immigr Minor Health.

[6] Goldenberg SM (2015). Trafficking, migration, and health: complexities and future directions. Lancet Global Health.

[7] Steen R, Jana S, Reza-Paul S, Richter M (2015). Trafficking, sex work, and HIV: efforts to resolve conflicts. Lancet.

[8] Ottisova L, Hemmings S, Howard LM, Zimmerman C, Oram S (2016). Prevalence and risk of Violence and the mental, physical and sexual health problems associated with human trafficking: an updated systematic review. Epidemiol Psychiatric Sci.

[9] ILO (2015). ILO Global Estimates on Migrant Workers and migrant domestic workers: results and methodology.

[10] de Lima P, Wright S (2009). Welcoming migrants? Migrant labour in Rural Scotland. Social Policy and Society.

[11] Aryal N, Regmi PR, van Teijlingen E, Simkhada P, Adhikary P, Bhatta YKD, Mann S. Injury and Mortality in Young Nepalese migrant workers: a call for Public Health Action. Asia Pac J Public Health. 2016. 10.1177/1010539516668628 27634831

[12] Simon J, Kiss N, Laszewska A, Mayer S (2015). Public health aspects of migrant health: a review of the evidence on health status for labour migrants in the European Region.

[13] Recknor F, Di Ruggiero E, Jensen E (2022). Addressing human trafficking as a public health issue. Can J Public Health.

[14] Boufkhed S, Thorogood N, Ariti C, Durand MA (2022). Building a better understanding of labour exploitation’s impact on migrant health: an operational framework. PLoS ONE.

[15] Sharma N (2017). The New Order of things’: immobility as protection in the regime of immigration controls. Anti-Trafficking Rev.

[16] Bryant K, Landman T. Combatting human trafficking since Palermo: what do we know about what works? J Hum Trafficking. 2020:1–22.

[17] Zimmerman C, Mak J, Pocock NS, Kiss L. Human trafficking: results of a 5-Year theory-based evaluation of interventions to prevent trafficking of women from South Asia. Front Public Health. 2021;9(400). 10.3389/fpubh.2021.645059 PMC8166201 34079782

[18] Kiss L, Zimmerman C (2019). Human trafficking and labor exploitation: toward identifying, implementing, and evaluating effective responses. PLoS Med.

[19] Pearhouse R (2008). Cambodia: human trafficking legislation threatens HIV response. HIV/AIDS Policy Law Review.

[20] Busza J, Castle S, Diarra A (2004). Trafficking and health. BMJ.

[21] Maynard R (2015). Fighting wrongs with wrongs? How Canadian anti-trafficking crusades have failed sex workers, migrants, and indigenous communities. Atlantis: Crit Stud Gend Cult Social Justice.

[22] Musto J, Fehrenbacher AE, Hoefinger H, Mai N, Macioti PG, Bennachie C et al. Anti-trafficking in the Time of FOSTA/SESTA: Networked Moral gentrification and sexual humanitarian creep. Social Sci [Internet]. 2021; 10(2).

[23] Collateral Damage, GAATW (2007). The impact of Anti-trafficking measures on Human rights around the World.

[24] Mende J (2019). The Concept of Modern Slavery: definition, Critique, and the Human rights Frame. Hum Rights Rev.

[25] Broad R, Turnbull N (2019). From human trafficking to modern slavery: the development of anti-trafficking policy in the UK. Eur J Criminal Policy Res.

[26] UNODC (2015). The role of recruitment fees and Abusive and Fraudulent Recruitment Practices of Recruitment Agencies in trafficking in persons.

[27] ILO (2015). Regulating Labour Recruitment To Prevent Human Trafficking and to Foster Fair Migration.

[28] McAlpine AM (2021). Mediated labour migration in the Myanmar-Thailand corridor and precarious outcomes: a mixed methods social network analysis and agent-based model.

[29] Fabbri C, Stöckl H, Jones K, Cook H, Galez-Davis C, Grant N (2023). Labor Recruitment and Human Trafficking: analysis of a global trafficking Survivor Database. Int Migrat Rev.

[30] Pocock NS, Stöckl H, Tadee R, Rongrongmuang W, Tharawan K, Zimmerman FBA (2021). Victims or suspects? Identifying and assisting potentially trafficked fishermen: a qualitative study with stakeholders and first responders in Thailand. J Migr Health.

[31] Protocol to Prevent, Suppress and Punish Trafficking in Persons, Especially Women and Children., (2000).

[32] Kern A, Müller-Böker U (2015). The middle space of migration: a case study on brokerage and recruitment agencies in Nepal. Geoforum.

[33] Awumbila M, Deshingkar P, Kandilige L, Teye JK, Setrana M (2017). Brokerage in migrant domestic work in Ghana: Complex Social relations and mixed outcomes.

[34] Ethiopia I (2017). Promote effective labour governance in Ethiopia: Program Achievements.

[35] Ayalew M, Aklessa G, Laiboni N (2019). Women’s Labour Migration on the African-Middle East Corridor: experiences of migrant domestic workers from Ethiopia.

[36] Zewdu GA (2018). Ethiopian female domestic labour migration to the Middle East: patterns, trends, and drivers. Afr Black Diaspora: Int J.

[37] Ayalew M, Minaye A (2017). Mental health status of returnee Ethiopian women from the Middle East vis-a-vis women in the process of migration: implication for intervention. EC Psychol Psychiatry.

[38] Habtamu K, Minaye A, Zeleke WA (2017). Prevalence and associated factors of common mental disorders among Ethiopian migrant returnees from the Middle East and South Africa. BMC Psychiatry.

[39] Fernandez B (2018). Health inequities faced by Ethiopian migrant domestic workers in Lebanon. Health Place.

[40] Reda AH (2018). An investigation into the experiences of female victims of trafficking in Ethiopia. Afr Black Diaspora: Int J.

[41] Demissie F (2018). Ethiopian female domestic workers in the Middle East and Gulf states: an introduction. Afr Black Diaspora: Int J.

[42] ILO (2017). The Ethiopian Overseas Employment Proclamation No.923/2016: a comprehensive analysis.

[43] Gazette FN (2020). Prevention and suppression of trafficking in persons and Smuggling of persons Proclamation 1178/2020.

[44] US Department of State (2023). Trafficking in persons Report.

[45] US Department of State. 2023 Trafficking in Persons Report: Ethiopia Washington, D.C.: Office to Monitor and Combat Trafficking in Persons; 2023 [Available from: https://www.state.gov/reports/2023-trafficking-in-persons-report/ethiopia/.

[46] Shewamene Z, Zimmerman C, Hailu E, Negeri L, Erulkar A, Anderson E et al. Migrant Women’s Health and Safety: Why Do Ethiopian Women Choose Irregular Migration to the Middle East for Domestic Work? Int J Environ Res Public Health [Internet]. 2022; 19(20). 10.3390/ijerph192013085 PMC9603558 36293665

[47] Meraki Labs. Protection Context for migrants passing through Yemen. Addis Ababa; 2019.

[48] Bisong A (2021). Regional solutions: regulating recruitment and protection of African migrant workers in the Gulf and Middle East.

[49] Adugna F, Deshingkar P, Ayalew T. Brokers, migrants and the state: Berri Kefach door operners in Ethiopian clandestine migration to South Africa. Brighton: University of Sussex; 2019. Contract No.: Working Paper 15.

[50] Schapendonk J (2018). Navigating the migration industry: migrants moving through an african-european web of facilitation/control. J Ethnic Migration Stud.

[51] Fernandez B (2013). Traffickers, brokers, employment agents and social networks: the regulation of intermediaries in the migration of Ethiopian domestic workers to the Middle East. Int Migrat Rev.

[52] Intertwined, ILO (2016). A study of employers of migrant domestic workers in Lebanon.

[53] Kiss L, Fotheringhame D, Mak J, McAlpine A, Zimmerman C. The use of bayesian networks for realist evaluation of complex interventions: evidence for prevention of human trafficking. J Comput Social Sci. 2020.

[54] Viuhko M (2018). Hardened professional criminals, or just friends and relatives? The diversity of offenders in human trafficking. Int J Comp Appl Criminal Justice.

[55] Alpes MJ. Why aspiring migrants trust migration brokers: the moral economy of departure in Anglophone Cameroon. Africa: Journal of the International African Institute. 2017;87(2):304 – 21.

[56] Skrivankova K (2010). Between decent work and forced labour: explorting the continuum of exploitation.

[57] Atnafu A, Adamek ME. The return migration experiences of Ethiopian women trafficked to Bahrain '... for richer or poorer, let me be on the hands of my people. African and Black Diaspora: An International Journal. 2016;9(2):243 – 56. – 21.

[58] Beck DC, Choi KR, Munro-Kramer ML, Lori JR, Trauma (2016). Violence & Abuse.

[59] Choi KR, Beck DC, Khan MA, Bell SA, Beza L, Munro-Kramer ML (2020). A qualitative needs assessment of human trafficking in Ethiopia: recommendations for a comprehensive, coordinated response. Int J Equity Health.

[60] Gezie LD, Worku A, Kebede Y, Gebeyehu A (2019). Sexual Violence at each stage of human trafficking cycle and associated factors: a retrospective cohort study on Ethiopian female returnees via three major trafficking corridors. BMJ Open.

[61] Lindquist J, Xiang B, Yeoh BSA (2012). Opening the black box of migration: brokers, the organization of transnational mobility and the changing political economy of Asia. Pac Affairs.

[62] Schwerdtle P, Bowen K, McMichael C (2018). The health impacts of climate-related migration. BMC Med.

[63] The Internal Displacement Monitoring Centre (2023). Global report on Internal Displacement 2023.

[64] Nyberg Sorenson N. Climate change, mobility and human trafficking in Ethiopia. Danish Institute for International Studies; 2023.

